# Comparative growth kinetics of *Listeria monocytogenes* and *Salmonella enterica* on dehydrated enoki and wood ear mushrooms during rehydration and storage

**DOI:** 10.3389/fmicb.2024.1406971

**Published:** 2024-08-05

**Authors:** Joelle K. Salazar, Josephina George, Megan L. Fay, Diana S. Stewart, David T. Ingram

**Affiliations:** ^1^Division of Food Processing Science and Technology, U.S. Food and Drug Administration, Bedford Park, IL, United States; ^2^Department of Food Science and Nutrition, Institute for Food Safety and Health, Illinois Institute of Technology, Bedford Park, IL, United States; ^3^Division of Produce Safety, U.S. Food and Drug Administration, College Park, MD, United States

**Keywords:** dried mushrooms, growth rates, *Listeria*, *salmonella*, survival

## Abstract

Specialty mushrooms have been implicated in foodborne illness outbreaks in the U.S. in recent years. These mushrooms are available to consumers in both their fresh and dried states. Dehydrating mushrooms is a convenient way to increase shelf life. The dehydration process results in a lowered water activity (a_w_) of the commodity, creating an environment where both spoilage and pathogenic bacteria cannot proliferate. Prior to food preparation and consumption, these mushrooms are typically rehydrated and possibly stored for later use which could lead to increased levels of pathogens. This study examined the survival and growth of *Listeria monocytogenes* and *Salmonella enterica* on dehydrated enoki and wood ear mushrooms during rehydration and subsequent storage. Mushrooms were heat dehydrated, inoculated at 3 log CFU/g, and rehydrated at either 5 or 25°C for 2 h. Rehydrated mushrooms were stored at 5, 10, or 25°C for up to 14 d. *L. monocytogenes* and *S. enterica* survived on enoki and wood ear mushroom types during rehydration at 5 and 25°C, with populations often <2.39 log CFU/g. During subsequent storage, no growth was observed on wood ear mushrooms, regardless of the rehydration or storage temperature, with populations remaining <2.39 log CFU/g for both pathogens. When stored at 5°C, no growth was observed for either pathogen on enoki mushrooms. During storage at 10 and 25°C, pathogen growth rates and populations after 14 d were generally significantly higher on the enoki mushrooms rehydrated at 25°C; the highest growth rate (3.56 ± 0.75 log CFU/g/d) and population (9.48 ± 0.62 log CFU/g) after 14 d for either pathogen was observed by *S. enterica* at 25°C storage temperature. Results indicate a marked difference in pathogen survival and proliferation on the two specialty mushrooms examined in this study and highlight the need for individual product assessments. Data can be used to assist in informing guidelines for time and temperature control for the safety of rehydrated mushrooms.

## 1 Introduction

Edible specialty mushrooms are common in the cuisines of Japan, China, and the Republic of Korea and are often consumed in soups, hot pots, and stir fry dishes. These specialty mushrooms have grown in popularity in the United States as more global cuisines are embraced by consumers. However, recent multistate foodborne illness outbreaks have occurred in the U.S. associated with two types of imported specialty mushrooms, enoki and wood ear (CDC, 2020a,b; FDA, 2020a,b; CDC, 2023). Firstly, fresh enoki mushrooms were the subject of two listeriosis outbreaks in 2020 and 2023 (CDC, 2020a; FDA, 2020a; CDC, 2023). The former outbreak resulted in 36 illnesses across 17 states, 31 hospitalizations, and four deaths (CDC, 2020a; FDA, 2020a), and the latter outbreak resulted in five illnesses and hospitalizations across four states (CDC, 2023). The enoki mushrooms implicated in these outbreaks were imported from Asian countries, including the Republic of Korea and China, and sold to consumers via retail establishments. Secondly, dried wood ear mushrooms were the subject of a salmonellosis outbreak in 2020, resulting in 55 illnesses across 12 states and six hospitalizations (CDC, 2020b; FDA, 2020b). The implicated dried wood ear mushrooms were sold directly to restaurants and, at least in some instances, were used as an ingredient in ramen soup (CDC, 2020b).

Specialty mushrooms, including enoki and wood ear, are commercially available to consumers in both their fresh and dried states. The shelf life of fresh mushrooms, like other fresh fruits and vegetables, is approximately 3–12 days depending on the packaging and storage conditions (Diamantopoulou and Philippoussis, 2015). One method commonly used to prolong the shelf life of mushrooms is drying or dehydration. Dried mushrooms have water activities (a_w_) <0.70 which prevents the proliferation of foodborne pathogens, if present. Previous research has determined that both *L. monocytogenes* and *S. enterica* can survive on dehydrated enoki and wood ear mushrooms stored for 180 d at 25°C (Fay et al., 2023). With an initial inoculation level of 6 log CFU/g, populations were reduced by approximately 2 and 4 log CFU/g on wood ear and enoki mushrooms during storage, respectively; however, both pathogens remained detectable via enrichment on the mushrooms after 180 d of storage. Similarly, *L. monocytogenes* and *S. enterica* are known to persist for long periods of time in other food products, including nuts and seeds, spices, flour, and confectionaries, often with no population reductions (Komitopoulou and Penaloza, 2009; Blessington et al., 2012; Farakos et al., 2017; Nascimento et al., 2018; Taylor et al., 2018).

If *L. monocytogenes* or *S. enterica* are present on the dried mushrooms prior to rehydration or incorporation into another food product with high a_w_, it is possible that the pathogens could survive or proliferate in the more favorable environments. For example, previous research has determined that *L. monocytognees* and *S. enterica* could survive on dehydrated vegetables, including carrot, corn, onion, pepper, and potato, during rehydration at 5 or 25°C and subsequent storage at 5, 10, or 25°C (Fay et al., 2023). In some cases, the growth rates during storage were as high as 2.37 and 1.63 log CFU/g/d for *L. monocytogenes* on potato and *S. enterica* on carrot, respectively, when the vegetables were rehydrated at 5°C and stored at 25°C. Lower pathogen growth rates were generally observed when the dehydrated vegetables were rehydrated at 5°C and then stored at 5°C. Results highlight the importance of holding rehydrated vegetables at refrigeration temperatures to reduce the potential for pathogen proliferation.

The objective of this study was to examine the survival of *L. monocytogenes* and *S. enterica* on dehydrated enoki and wood ear mushrooms during both rehydration and subsequent storage at different temperatures. Two different rehydration temperatures (5 and 25°C) and three different storage temperatures (5, 10, and 25°C) were employed in the study. The FDA Food Code currently lists certain fresh cut produce, including cut leafy greens, cut tomatoes, and cut melon, as foods requiring time and temperature control for safety (TCS foods) (FDA, 2022). However, there currently are no time and temperature guidelines for safety for the rehydration and storage of previously-dehydrated foods, including mushrooms. The results from this study will provide information on the time and temperature combinations for rehydration and storage for the safety of these two specialty mushrooms.

## 2 Materials and methods

### 2.1 Mushroom selection, preparation, and dehydration

Fresh raw enoki and wood ear mushrooms were purchased from local grocers in Illinois (United States) and stored at 5°C for up to 24 h prior to dehydration. For enoki mushrooms, the bottom 3 cm containing the substrate was discarded and mushrooms were chopped into 2.5 cm long pieces. For wood ear, mushrooms were chopped into 2.5 × 2.5 cm pieces. Chopped mushrooms were arranged in a single layer on wax paper and dehydrated at 60°C for up to 24 h in a commercial food dehydrator (Excalibur model EXC10EL, The Legacy Companies, Weston, FL, United States). Triplicate 10-g mushroom samples were removed from the dehydrator after 0, 1, 2, 3, 4, 6, and 24 h for moisture content analysis (see section 2.5). After dehydration, mushrooms were stored in sealed bags for up to 24 h.

### 2.2 Strains and culture conditions

A four-strain cocktail of either *Salmonella enterica* or *Listeria monocytogenes* was used in this study. The *S. enterica* strains consisted of Enteritidis PT30 (ATCC BAA-1045), Agona 447967, Typhimurium 46249 (cantaloupe outbreak isolate), and Newport 36796 (CFSAN046260, tomato outbreak isolate). For *L. monocytogenes*, the strains used were LS806 (isolated from hummus), LS3132 (isolated from avocado), LS1863 (FDA1142659-C001-001, enoki mushroom outbreak isolate), and ScottA. All of the *L. monocytogenes* strains used were rifampicin resistant (100 μg/mL); spontaneous rifampicin resistant variants were obtained by successive culturing with increasing concentrations of rifampicin, up to 100 μg/mL. Strains were cultured individually in Tryptic Soy Broth (TSB; Becton, Dickinson and Co., Sparks, MD) for 16–18 h at 37°C. Cultures were washed twice with Butterfields’s Phosphate Buffer (BPB, pH 7.2) and combined in equal volumes to create a four-strain cocktail of either *S. enterica* or *L. monocytogenes* (9 log CFU/mL). The cocktails were serially diluted in BPB and plated onto Tryptic Soy Agar (TSA; Becton, Dickinson and Co.) to verify the initial population levels; plates were incubated at 37°C for 24 h prior to enumeration.

### 2.3 Inoculation of dehydrated mushrooms

Dehydrated mushrooms (200 g) were inoculated with a 5 mL of a diluted cocktail of either *S. enterica* or *L. monocytogenes* in a 3-L stomacher bag, resulting in approximately 3 log CFU/g. The bag was shaken by hand for 5 min to evenly distribute the inoculum. Inoculated mushrooms were spread into a single layer on a foil tray and dried at ambient temperature in a biosafety cabinet for 1 h with the blower on. Triplicate 10-g mushroom samples after inoculation and after 1 h drying were used for moisture content analysis (see section 2.5) and pathogen enumeration (see section 2.6).

### 2.4 Mushroom rehydration and storage

Dehydrated mushrooms (approximately 170 g) were rehydrated 1:20 with water in sterile metal bowls for 2 h. The water and air temperature during rehydration was maintained at either 5 or 25°C. After 0, 5, 15, 30, 60, 90, and 120 min of rehydration, approximately 50-g of mushrooms were removed from the water and strained for 10 min. Triplicate 10-g samples were used for pathogen enumeration (see section 2.6) and duplicate 10-g samples were used for moisture content and water activity analysis (see section 2.5). After 2 h of rehydration, the remaining mushrooms were strained for 10 min using a colander. After draining, mushrooms were portioned into 8-oz. deli containers with lids (40 g each) and stored at 5, 10, or 25°C for up to 14 d. After 0, 1, 3, 6, 9, and 14 d, both *L. monocytogenes* and *S. enterica* were enumerated from triplicate 10-g samples (see section 2.6). Three independent trials for each pathogen-mushroom-rehydration temperature combination were conducted in triplicate (*n* = 9).

### 2.5 Measurement of water activity (a_w_) and moisture content

To measure a_w_, a 1-g sample of inoculated mushroom was measured using an a_w_ meter (Aqualab 4TE, Meter Group, Pullman, WA, United States). For moisture content, a 10-g sample of inoculation mushroom was placed into an oven at 100°C for 24 h. The solid weight after 24 h was measured and the moisture content (wet basis) was then calculated based on the initial weight.

### 2.6 Enumeration of *L. monocytogenes* and *S. enterica*

Mushroom samples (10 g) were combined 1:10 with either Buffered *Listeria* Enrichment Broth (BLEB; Becton, Dickinson and Co., Sparks, MD, United States) or BPB for *L. monocytogenes* or *S. enterica*, respectively, and homogenized using a stomacher (Stomacher 400C Circulator, Seward Laboratory Systems Inc., Bohemia, NY) for 1 min. Homogenates were serially diluted and plated onto Brain Heart Infusion Agar (BHIA; Becton, Dickinson and Co., Sparks, MD, United States) with 100 μg/mL of rifampicin (BHIA^rif^) or onto TSA with Xylose Lysine Deoxycholate (XLD; Becton, Dickinson and Co., Sparks, MD, United States) agar overlay for enumeration of *L. monocytogenes* or *S. enterica*, respectively. Agar plates were incubated at 37°C for 24–48 h. Data were expressed as log CFU/g. The lower limit of enumeration of the plate count assay (i.e., 25 CFU/plate) was 2.39 log CFU/g. When pathogen populations were expected to be below the limit of enumeration, samples were enriched according to the FDA Bacteriological Analytical Manual (FDA, 2017).

### 2.7 Primary modeling and statistical analysis

The growth kinetics (growth rates and lag phases) of both *L. monocytogenes* and *S. enterica* on rehydrated enoki mushrooms during 14 d storage at 5, 10, or 25°C were determined using the DMFit v 3.0 add-in for Excel using the Baranyi and Roberts primary model (Baranyi and Roberts, 1994). Differences between growth rates were statistically analyzed using ANCOVA with Tukey’s *post hoc* test. Differences in a_w_, moisture contents, and populations were statistically analyzed using ANOVA with Tukey’s *post hoc* test. Differences in the number of wood ear mushroom samples where *L. monocytogenes* or *S. enterica* was detected were determined using Fisher’s Exact test. *p* ≤ 0.05 was considered significant.

## 3 Results

### 3.1 Properties of the fresh, dehydrated, and rehydrated mushrooms

The a_w_ and moisture contents (wet basis) of the fresh enoki and wood ear mushrooms have been previously reported (Fay et al., 2023). The a_w_ values were 0.974 ± 0.012 and 0.976 ± 0.008 and the moisture contents were 89.34 ± 0.98 and 88.60 ± 0.55%, respectively. The a_w_ and moisture contents of both mushroom types after dehydration, after inoculation, and after drying of the inoculum were determined in this study (Table 1). After dehydration at 60°C for 24 h, the a_w_ and moisture contents of both mushroom types significantly decreased; the a_w_ of the dehydrated enoki and wood ear mushrooms were 0.209 ± 0.002 and 0.186 ± 0.026 and the moisture contents were 4.50 ± 2.00 and 4.91 ± 2.73%, respectively. No difference was determined between the two mushroom types. After inoculation with either the *L. monocytogenes* or *S. enterica* cocktail, the a_w_ of both mushroom types significantly increased, while no significant differences in the moisture contents was observed. After inoculation, the a_w_ of the wood ear mushrooms (0.537 ± 0.099) was higher than that of enoki (0.302 ± 0.066). However, after the inoculum was allowed to dry on the mushrooms for 1 h, no significant difference in values was observed between the enoki and wood ear a_w_ values. The a_w_ of both the wood ear and enoki mushrooms were significantly different after drying (0.287 ± 0.062 and 0.313 ± 0.024, respectively) compared to pre-inoculation (0.186 ± 0.026 and 0.209 ± 0.002, respectively).

**Table 1:** The water activity (a_w_) and moisture content (%) of the enoki and wood ear mushrooms after dehydration, after inoculation, and after drying.

Mushroom	a_w_			Moisture (%)		
	After dehydration^a^	After inoculation	After drying^2^	After dehydration^b^	After inoculation	After drying^2^
Enoki	0.209 ± 0.002^aA^	0.302 ± 0.066^aB^	0.313 ± 0.024^aB^	4.50 ± 2.00^aA^	7.51 ± 2.37^aA^	8.72 ± 1.87^aA^
Wood ear	0.186 ± 0.026^aA^	0.537 ± 0.099^bB^	0.287 ± 0.062^aC^	4.91 ± 2.73^aA^	7.87 ± 2.91^aA^	7.70 ± 1.40^aA^

^a^ After dehydration at 60°C for 24 h; ^b^ after 1 h of inoculum drying. Different lowercase letters indicate significant difference between the water activity or moisture content of enoki and wood ear mushrooms at the same time (columns). Different uppercase letters indicate significant difference between the between the water activity or moisture content of enoki or wood ear mushrooms at different times (rows).

**Figure 1:**
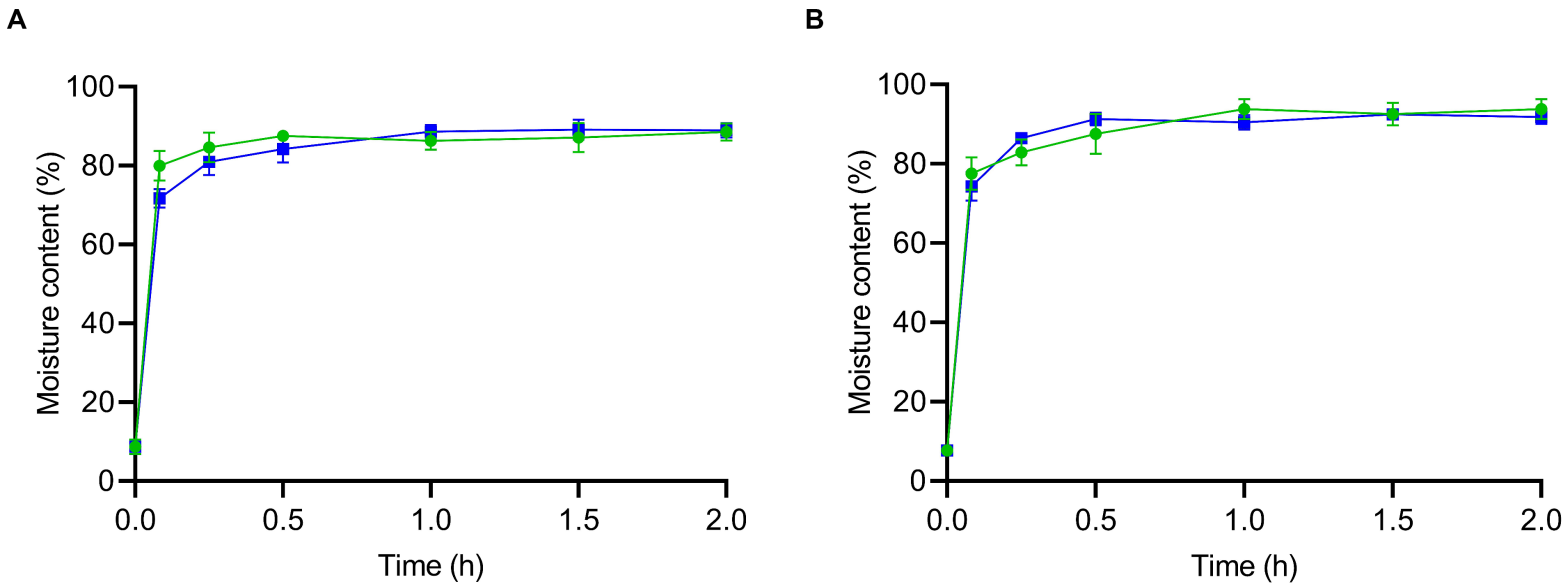
The moisture content (%) of the enoki **(A)** and wood ear **(B)** mushrooms during rehydration at 5 (green circles) or 25°C (blue squares) for 2 h. Data are mean values ± standard deviation (*n* = 9).

The moisture content of both mushroom types was monitored during rehydration at either 5 or 25°C for 2 h (Figure 1). Prior to rehydration (after drying the inoculum for 1 h), the moisture contents of the enoki and wood ear mushrooms were 8.72 ± 1.87 and 7.70 ± 1.40%, respectively. A significant increase in moisture content for both mushroom types occurred after only 5 min of rehydration: for enoki mushrooms, moisture contents were 79.96 ± 3.79 and 71.73 ± 2.39% at 5 and 25°C, respectively, which were significantly different; the moisture contents for the wood ear mushrooms were 77.50 ± 4.10 and 74.25 ± 3.59%, respectively.

### 3.2 Survival of *L. monocytogenes* and *S. enterica* on dehydrated mushrooms during rehydration

The populations of *L. monocytogenes* and *S. enterica* after inoculation, after drying of the inoculum for 1 h, and after rehydration at 5 or 25°C for 2 h are presented in Table 2. The initial inoculation levels of both pathogens on both mushroom types were not significantly different and ranged from 2.73 ± 0.02 to 3.27 ± 0.27 log CFU/g. No significant difference in populations were observed on the mushrooms after drying the inocula for 1 h. However, a significantly lower population of *L. monocytogenes* was observed on the wood ear mushrooms (2.58 ± 0.15 log CFU/g) after drying compared to on the enoki (3.22 ± 0.26 log CFU/g). After inoculum drying, both mushroom types were rehydrated at either 5 or 25°C for 2 h. Both pathogens survived on both mushroom types after rehydration regardless of the temperature. After rehydration at 5°C, both pathogen populations were < 2.39 log CFU/g on both mushroom types; both pathogens remained detectable on both mushroom types via enrichments. After rehydration at 25°C, pathogen populations on enoki mushrooms were 2.65 (8/9 values <2.39 log CFU/g) and 2.78 ± 0.27 log CFU/g for *L. monocytogenes* and *S. enterica*, respectively. On wood ear mushrooms, the populations of both pathogens were < 2.39 log CFU/g but remained detectable via enrichments.

**Table 2:** Populations of *L. monocytogenes* and *S. enterica* on dehydrated enoki and wood ear mushrooms after inoculation, after drying, and after rehydration at 5 or 25°C.

Pathogen	Mushroom	Population (log CFU/g±SD^a^)			
		After inoculation	After drying^b^	After rehydration^c^	
				5°C	25°C
*L. monocytogenes*	Enoki	3.27 ± 0.27^aA^	3.22 ± 0.26^aA^	<2.39	2.65^d^
	Wood ear	3.13 ± 0.35^aA^	2.58 ± 0.15^bA^	<2.39	<2.39
*S. enterica*	Enoki	2.79 ± 0.24^aA^	2.80 ± 0.25^abA^	<2.39	2.78 ± 0.27^A^
	Wood ear	2.73 ± 0.02^aA^	2.76 ± 0.02^bA^	<2.39	<2.39

^a^ Standard deviation; ^b^ after 1 h of inoculum drying; ^c^ after 2 h rehydration at either 5 or 25°C (water and air temperature); ^d^ 8/9 values <2.39 log CFU/g. Different lowercase letters indicate significant difference between populations on enoki and wood ear mushrooms at the same time (columns). Different uppercase letters indicate significant difference between the populations on enoki or wood ear mushrooms at different times (rows).

### 3.3 Dynamics of *L. monocytogenes* and *S. enterica* on rehydrated enoki mushrooms

**Figure 2:**
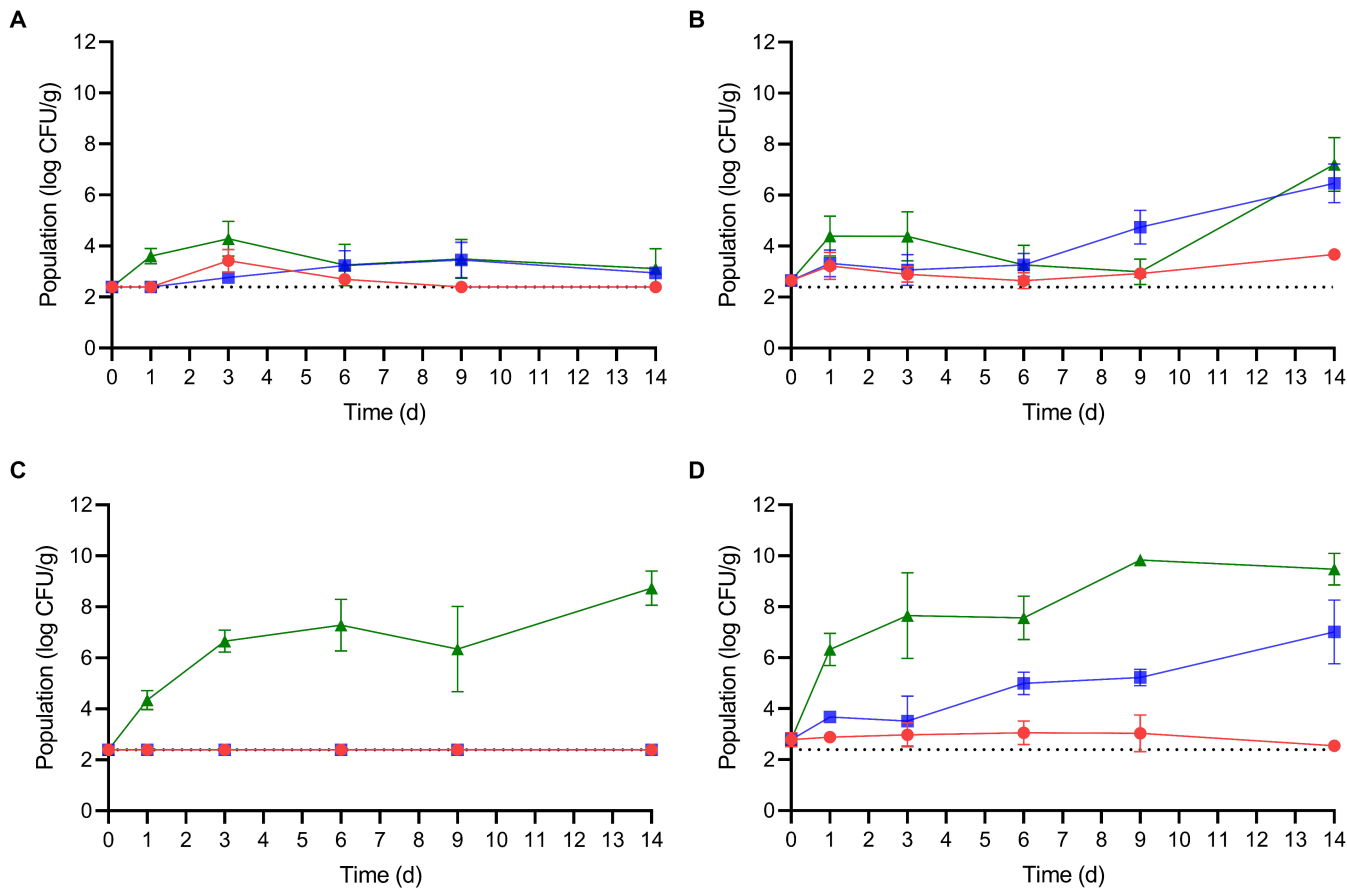
The population dynamics of *L. monocytogenes* **(A,B)** and *S. enterica* **(C,D)** on rehydrated enoki mushrooms during storage at 5 (red circles), 10 (blue squares) or 25°C (green triangles) for 14 days. Mushrooms were rehydrated at either 5 **(A,C)** or 25°C **(B,D)**. Data are mean values ± standard deviation (*n* = 9). The dotted horizontal line indicates the lower level of enumeration (2.39 log CFU/g).

After the enoki mushrooms were rehydrated at either 5 or 25°C for 2 h, the mushrooms were stored at 5, 10, or 25°C for up to 14 d. The population dynamics of *L. monocytogenes* and *S. enterica* on rehydrated enoki mushrooms during storage are displayed in Figure 2. Specific growth kinetics, including growth rates, lag phases, and times for a 1 log CFU/g population increase are presented in Table 3. When enoki mushrooms were rehydrated at 5°C and stored at 5°C, *L. monocytogenes* survived but did not increase in population during storage; the population after 14 d was <2.39 log CFU/g. When rehydrated at 5°C and stored at the higher temperatures, 10 and 25°C, *L. monocytogenes* proliferated on the enoki mushrooms with growth rates of 0.17 ± 0.11 and 1.22 ± 0.55 log CFU/g/d, respectively, resulting in 1 log CFU/g increases in 5.88 and 0.82 d. While no lag phases were predicted for *L. monocytogenes* on enoki mushrooms rehydrated at 5°C, a lag phase of 5.67 ± 1.39 d was predicted when rehydrated at 25°C and stored at 10°C. Both the growth rate and population of *L. monocytogenes* (0.42 ± 0.08 log CFU/g/d and 6.47 ± 0.76 log CFU/g, respectively) on the enoki mushrooms rehydrated at 25°C and stored at 10°C were significantly higher compared to when rehydration occurred at 5°C. The growth rate of *L. monocytogenes* on the enoki mushrooms rehydrated at 25°C and stored at 25°C was 1.75 ± 0.84 log CFU/g/d, resulting in a 1 log CFU/g population increase in only 0.57 d. The population after 14 d was 7.21 ± 1.05 log CFU/g, which was the highest population attained by *L. monocytogenes*.

**Table 3:** Growth kinetics of *L. monocytogenes* and *S. enterica* on rehydrated enoki mushrooms during subsequent storage at 5, 10, or 25°C for 14 days.

Pathogen	Rehydration temperature (°C)^a^	Storage temperature (°C)	Growth rate (log CFU/g/d ± SE^b^)	Lag (d ± SE)	*r* ^2 c^	Time (d) to a 1 log CFU/g increase^d^	Population after 14 d storage (log CFU/g ± SD^e^)
*L. monocytogenes*	5	10	0.17 ± 0.11^ab^	ND^f^	0.47	5.88	2.94 ± 0.24^a^
		25	1.22 ± 0.55^c^	ND	0.51	0.82	3.11 ± 0.78^ab^
	25	10	0.42 ± 0.08^d^	5.67 ± 1.39	0.81	8.05	6.47 ± 0.76^c^
		25	1.75 ± 0.84^c^	ND	0.78	0.57	7.21 ± 1.05^cd^
*S. enterica*	5	25	1.27 ± 0.27^c^	ND	0.63	0.79	8.73 ± 0.68^de^
	25	10	0.28 ± 0.03^bd^	ND	0.76	3.57	7.02 ± 1.25^cd^
		25	3.56 ± 0.75^e^	ND	0.74	0.28	9.48 ± 0.62^e^

^a^ Water and air temperature; ^b^ standard error; ^c^ *r*^2^, coefficient of determination; ^d^ calculated based on growth rate and lag phase (if applicable); ^e^ standard deviation; ^f^ none determined. Different lowercase letters indicate significant difference between growth rates or populations (columns).

When enoki mushrooms were rehydrated at 5°C and stored at 5 or 10°C, *S. enterica* survived but remained <2.39 log CFU/g during storage. When rehydrated at 5°C and stored at 25°C, *S. enterica* proliferated on the enoki mushrooms with a growth rate of 1.27 ± 0.27 log CFU/g/d, resulting in a 1 log CFU/g population increase in only 0.79 d. The population after 14 d of storage at 25°C was 8.73 ± 0.68 log CFU/g. *S. enterica* survived but did not grow on enoki mushrooms rehydrated at 25°C and stored at 5°C, with a population after 14 d of 2.54 log CFU/g (8/9 values <2.39 log CFU/g). The pathogen proliferated on enoki mushrooms rehydrated at 25°C and stored at 10°C, with a growth rate of 0.28 ± 0.03 log CFU/g/d, resulting in 1 log CFU/g population increases in 3.57 d, respectively. The highest growth rate of either pathogen on enoki mushrooms was observed by *S. enterica* at 25°C, 3.57 ± 0.75 log CFU/g/d, resulting in a 1 log CFU/g population increase in only 0.28 d. The population of *S. enterica* after 14 d storage at 25°C was 9.48 ± 0.62 log CFU/g, which was the highest population attained by either pathogen on enoki mushrooms.

### 3.4 Survival of *L. monocytogenes* and *S. enterica* on rehydrated wood ear mushrooms

After the wood ear mushrooms were rehydrated at either 5 or 25°C for 2 h, the mushrooms were stored at 5, 10, or 25°C for up to 14 d. Both *L. monocytogenes* and *S. enterica* populations remained <2.39 log CFU/g during storage, regardless of the rehydration or storage temperature. Table 4 displays the number of wood ear mushroom samples where *L. monocytogenes* or *S. enterica* were detected (*n*/9) via enrichment after 1, 7, and 14 d of storage at 5, 10, or 25°C. After 1 d of storage, *L. monocytogenes* was detected in 100% (9/9) of samples regardless of the rehydration or storage temperature. *S. enterica* was detected in 100% (9/9) of samples at all storage temperatures when wood ear mushrooms were rehydrated at 25°C, however, was detected in 78% (7/9) of samples when rehydrated at 5°C and stored at 10 or 25°C. After 7 d of storage, no significant difference in detection occurred with either pathogen, ranging from 100% (9/9) of samples for *L monocytogenes* on wood ear mushrooms rehydrated at 25°C and stored at 5 or 10°C to 56% (5/9) of samples for *S. enterica* on mushrooms rehydrated at 5°C and stored at 10°C. After 14 d of storage, *L. monocytogenes* detection was lowest on wood ear mushrooms stored at 25°C (33% (3/9) and 56% (5/9) of samples when mushrooms were rehydrated at 5 and 25°C, respectively). Detection of *S. enterica* was lowest on wood ear mushrooms rehydrated at 25°C and stored at 10 or 25°C (44% (4/9) of samples).

**Table 4:** Detection of *L. monocytogenes* and *S. enterica* on rehydrated wood ear mushrooms during subsequent storage at 5, 10, or 25°C for 14 days.

Pathogen	Rehydration temperature (°C)^a^	Storage temperature (°C)	The # of samples where the pathogen was detected (*n*/9)		
			1 d	7 d	14 d
*L. monocytogenes*	5	5	9^aA^	8^aA^	9^aA^
		10	9^aA^	6^aA^	9^aA^
		25	9^aA^	7^aA^	3^bB^
	25	5	9^aA^	9^aA^	9^aA^
		10	9^aA^	9^aA^	9^aA^
		25	9^aA^	8^aA^	5^abB^
*S. enterica*	5	5	9^aA^	9^aA^	8^aA^
		10	7^aA^	5^aA^	9^aA^
		25	7^aA^	6^aA^	8^aA^
	25	5	9^aA^	9^aA^	7^abA^
		10	9^aA^	6^aAB^	4^bB^
		25	9^aA^	7^aAB^	4^bB^

^a^ Water and air temperature. Different lowercase letters indicate significant difference between the number of wood ear samples (*n*/9) where *L. monocytogenes* or *S. enterica* was detected on the same day (columns). Different uppercase letters indicate significant difference between the number of wood ear samples (*n*/9) on different days where *L. monocytogenes* or *S. enterica* was detected when mushrooms were rehydrated and stored at the same temperature (rows).

## 4 Discussion

Recent outbreaks associated with enoki and wood ear mushrooms in the U.S. have prompted research to understand the fate of *L. monocytogenes* and *S. enterica* on these food products. Since the shelf life of fresh mushrooms is relatively short, mushrooms are often dried to extend their shelf life, reducing their a_w_. Prior to consumption, dried mushrooms could be rehydrated and stored for later use. Once dried mushrooms are rehydrated, the high moisture content and relatively neutral pH of these foods would provide an environment suitable for the proliferation of foodborne pathogens, and hence product assessments are needed. Therefore, this study examined the survival of *L. monocytogenes* and *S. enterica* on dehydrated enoki and wood ear mushrooms during rehydration and subsequent storage.

Two rehydration temperatures were incorporated in this study to understand if the temperature of rehydration influenced the survival or proliferation of either pathogen. Since consumers, retail establishments, and restaurants may rehydrate the dehydrated mushrooms in the refrigerator or at room temperature, these two conditions (5 and 25°C) were mimicked in this study. Both *L. monocytogenes* and *S. enterica* survived on both mushroom types during rehydration, while populations were often below quantification limits (<2.39 log CFU/g). Similar *L. monocytogenes* populations were observed in a previous study when dehydrated vegetables, including carrot, corn, onion, pepper, and potato, were rehydrated at 5 or 25°C (Fay et al., 2023). Similar *S. enterica* populations were also observed on the vegetables during rehydration at 5°C, although much higher populations (4–6 log CFU/g) were attained on certain vegetables (carrot, pepper, and potato) when rehydration occurred at 25°C. It is noted that a 24-h rehydration was used compared to the 2-h rehydration in this study, and results may be different if rehydration continued for longer than 2 h. The survival and/or proliferation of pathogens during rehydration at 5 or 25°C appears to be food matrix dependent.

After the dehydrated mushrooms were rehydrated, they were stored at 5, 10, or 25°C, mimicking refrigeration, temperature abuse, and room temperature, respectively. Striking differences were observed in the survival of both pathogens on the two mushroom types during storage with enoki mushrooms appearing to be a much more favorable environment. While both pathogens survived on enoki mushrooms stored at 5°C regardless of the rehydration temperature, both pathogens proliferated at 10 and 25°C with 1 log CFU/g population increases in 0.28 to 8.05 d. However, in the case of wood ear mushrooms, both pathogens survived during the 14-d storage period but did not grow, even at the higher storage temperatures. This difference may be attributed to antimicrobial compounds present in wood ear mushrooms, including polysaccharides, melanin, and polyphenols; these compounds have been shown to inhibit growth and biofilm formation of bacterial pathogens (Cai et al., 2015; Yu and Oh, 2016; Liu et al., 2021). While the antimicrobial aspect of wood ear mushrooms resulted in hindered growth of both pathogens, both pathogens remained detectable during storage.

The antimicrobial nature of the wood ear mushrooms may have also contributed to the significantly lower population of *L. monocytogenes* on these mushrooms when compared to the enoki mushrooms after drying of the inocula in this study. Interestingly, the antimicrobial nature of wood ear mushrooms may be more pronounced when the mushrooms are in their dehydrated state or rehydrated from dried, as previous research has shown that both *L. monocytogenes* and *S. enterica* can proliferate on fresh whole and chopped wood ear mushrooms (Fay et al., 2023). For example, *L. monocytogenes* grew on whole and chopped wood ear mushrooms at both 10 and 25°C, with 1 log CFU/g population increases in 33.33 to 1.82 d. *S. enterica* also proliferated on whole and chopped wood ear mushrooms, but only at 25°C, with a 1 log CFU/g population increase in 0.80 to 2.08 d. A similar phenomenon has been shown to occur with onions, also known to have antimicrobial properties (de Niederhäusern et al., 2021), where the growth rates of *L. monocytogenes* were significantly higher on fresh onion compared to dehydrated onion that had been rehydrated and stored at 10 or 25°C (Salazar et al., 2017; Fay et al., 2023).

The survival and growth of *L. monocytogenes* on the rehydrated enoki mushrooms in this study is comparable to that on fresh whole and chopped enoki mushrooms stored at both 10 and 25°C, with no significant difference observed in growth rates (Fay et al., 2023). For *S. enterica*, growth rates were generally similar at all storage temperatures on the fresh enoki and the enoki rehydrated at 25°C (Fay et al., 2023). However, *S. enterica* did not grow on the enoki rehydrated at 5°C and stored 10°C in this study, whereas 1 log CFU/g population increases were observed in 7.69 to 8.33 d on the fresh enoki stored at 10°C. These findings indicate that pathogen survival may be different on the same commodity depending on if it is fresh or rehydrated from a dehydrated state, further highlighting the need for specific product assessments.

This study evaluated the survival of *L. monocytogenes* and *S. enterica* on dehydrated enoki and wood ear mushrooms during rehydration and storage. Marked differences were observed in pathogen survival on these two rehydrated specialty mushrooms, emphasizing the need for individual assessments. Rehydrated enoki mushrooms appeared to be a more favorable environment for both pathogens and growth was hindered when rehydration occurred at 5°C and the mushrooms were stored at 5°C. The results therefore highlight the importance of storing rehydrated mushrooms at refrigeration temperatures. Data from this study can be used to inform regulatory decisions surrounding time and temperature control for safety for these food products.

## Data availability statement

The original contributions presented in the study are included in the article/supplementary material, further inquiries can be directed to the corresponding author.

## Author contributions

JS: Conceptualization, Data curation, Formal analysis, Investigation, Methodology, Supervision, Writing – original draft, Writing – review & editing. JG: Conceptualization, Data curation, Formal analysis, Investigation, Methodology, Visualization, Writing – original draft, Writing – review & editing. MF: Data curation, Formal analysis, Investigation, Methodology, Writing – original draft, Writing – review & editing. DS: Data curation, Investigation, Methodology, Writing – original draft, Writing – review & editing. DI: Conceptualization, Investigation, Writing – original draft, Writing – review & editing.
